# Retraction Note: Thymoquinone therapy remediates elevated brain tissue inflammatory mediators induced by chronic administration of food preservatives

**DOI:** 10.1038/s41598-026-41316-6

**Published:** 2026-03-11

**Authors:** Ahmed Mohsen Hamdan, Mohammed M. Al-Gayyar, Mohamed E. E. Shams, Udai Salamh Alshaman, Kousalya Prabahar, Alaa Bagalagel, Reem Diri, Ahmad O. Noor, Diena Almasri

**Affiliations:** 1 Department of Pharmacy Practice, Faculty of Pharmacy, University of Tabuk, Tabuk, Saudi Arabia; 2 Department of Pharmaceutical Chemistry, Faculty of Pharmacy, University of Tabuk, Tabuk, Saudi Arabia; 3 Department of Biochemistry, Faculty of Pharmacy, Mansoura University, Mansoura, Egypt; 4 Department of Pharmaceutics, Faculty of Pharmacy, Mansoura University, Mansoura, Egypt; 5 Department of Pharmaceutics and Pharmacy Practice, Oman College of Health Sciences, Pharmacy Program, Ministry of Health, Muscat, Oman; 6 Department of Pharmacy Practice, Faculty of Pharmacy, Kind Abdulaziz University, Jeddah, Saudi Arabia

Retraction of: *Scientific Reports* 10.1038/s41598-019-43568-x, published online 07 May 2019

The Editors have retracted this Article.

After publication, concerns were raised about the appropriateness of the model used in the study. The study aimed to explore the impact of the food preservatives (in particular sodium nitrate) and whether thymoquinone can have protective function against oxidative stress caused by chronic exposure to this chemical. However, the dosage of sodium nitrite used at the study was at the level of half of the LD_50_ for rats, which would model extreme rather than low-level exposure. The mismatch between the model and the research aims casts doubts on the conclusions of the Article. Additionally, concerns were also raised regarding the data presented in this Article.

- Figure 6C (Control group) and Figure 6D (TQ treated control group) appear to originate from the same sample.
- Figure 6E (SN group) and Figure 6E (TQ treated SN group) appears to have some partial overlap.

Authors were unable to provide the raw images and satisfactory explanation for raised concerns. The Editors therefore no longer have confidence in the research presented in this work.

Ahmed Mohsen Hamdan, Kousalya Prabahar, Mohamed E. E. Shams and Mohammed M. Al-Gayyar disagree with this retraction. None of the remaining Authors have responded to correspondence from the Publisher about this retraction.

